# Black Soldier Fly Larvae Bioconvert Deoxynivalenol-Contaminated Feed Without Toxin Accumulation: Growth Performance, Residue Distribution, and Gut Microbiota Responses

**DOI:** 10.3390/microorganisms14071452

**Published:** 2026-07-01

**Authors:** Kun Liu, Yuting Li, Minghui Jiao, Jianlai Guo, Xiangbo Ji, Huibin Shi, Jun Li, Weixian Zhang, Kai Quan, Zhentian Li, Xilan Jiao

**Affiliations:** 1 Henan Key Laboratory of Livestock and Poultry Genetic Improvement and Healthy Breeding, Henan University of Animal Husbandry and Economy, Zhengzhou 450046, China; 13837147053@163.com (J.G.); 80104@hnuahe.edu.cn (X.J.); huibinshi0715@163.com (H.S.); lijun.nn@163.com (J.L.); zhangwx126@126.com (W.Z.); quankai1115@163.com (K.Q.); 2 College of Animal Science and Technology, Henan Agricultural University, Zhengzhou 450046, China; liyuting018@163.com (Y.L.); m18239340797@163.com (M.J.); lizhentian2006@126.com (Z.L.); 3 College of Veterinary Medicine, Henan Agricultural University, Zhengzhou 450046, China

**Keywords:** deoxynivalenol, black soldier fly larvae, gut microbiota, detoxification, bioconversion

## Abstract

Deoxynivalenol (DON) contamination poses a major threat to feed safety and animal health, yet safe and sustainable strategies for managing DON-contaminated feed remain limited. Black soldier fly larvae (BSFL) have shown strong tolerance to various mycotoxin-contaminated substrates and generally exhibit limited toxin bioaccumulation, making them a promising biological system for the valorization of contaminated organic resources. This study evaluated the effects of DON-contaminated feed on BSFL growth performance, bioconversion efficiency, DON residue distribution, and gut microbial responses. Results showed that DON exposure had no significant effect on larval survival, body length, or body weight, nor on the efficiency of conversion of digested feed, substrate reduction rate, or waste reduction index. Residue analysis showed that DON was below the limit of detection in larval samples after BSFL treatment, while the DON concentration in frass was approximately 81.10% lower than that in the initial substrate, indicating no detectable *in vivo* bioaccumulation. Gut microbiota analysis showed no significant changes in alpha diversity, with minor compositional trends in specific taxa. *Firmicutes* remained the predominant phylum, whereas *Actinobacteriota* increased. These findings provide new insights into host–microbe adaptations under mycotoxin stress and support further evaluation of BSFL-based strategies for managing DON-contaminated feed resources.

## 1. Introduction

Deoxynivalenol (DON), also known as vomitoxin, is a type B trichothecene mycotoxin produced by *Fusarium* species, primarily during field infection by *Fusarium graminearum* [1,2,3]. Major cereal crops, including maize, wheat, and barley, are frequently contaminated with DON in North America and the Asia-Pacific region [1,4]. According to the dsm-firmenich World Mycotoxin Survey, DON showed a high prevalence in several major feed-producing regions, including Asia and North America, based on survey data from January to June 2023 and 2024 [5]. In animal production, ingestion of DON-contaminated feed can impair key metabolic organs, particularly the intestine and liver, and subsequently induce immune dysfunction and oxidative stress, thereby severely compromising animal health [6,7,8,9]. Given its potential risks and increasingly stringent regulatory limits, the development of safe and efficient DON detoxification or biodegradation strategies has become an important research priority for improving food and feed quality and safeguarding animal health.

Despite continuous advances in DON detoxification technologies, achieving effective and safe removal of DON from complex matrices remains challenging because of its high thermal and chemical stability. Conventional physical and chemical detoxification methods can reduce DON levels to some extent; however, their detoxification efficiency is often affected by matrix composition, and these methods may cause nutrient loss or pose risks of secondary pollution [10,11]. In contrast, biodegradation can convert DON into less toxic metabolites through the specific activities of microorganisms or enzymes, and is therefore considered a promising detoxification strategy because of its mild reaction conditions, strong selectivity, and environmental compatibility [12,13]. In recent years, several DON-degrading microorganisms have been isolated from the intestinal tracts of poultry and livestock, providing important microbial resources for DON biodetoxification [14,15]. However, the DON-degrading capacities of different biological sources and the associated changes in microbial communities remain insufficiently understood. Previous studies have shown that DON biodegradation can be influenced by multiple factors, including microbial composition, culture conditions, and matrix properties [16,17]. Therefore, exploring novel bioconversion systems and clarifying the relationship between DON reduction and microbial community shifts may provide new theoretical support and microbial resources for DON biodetoxification.

Against this background, black soldier fly larvae (BSFL), which possess complex substrate conversion capabilities and a rich gut microbiota, offer a novel direction for exploring DON bioconversion. BSFL exhibit exceptionally high conversion efficiency and environmental adaptability in the valorization of organic waste [18,19,20,21]. More importantly, the larvae demonstrate high tolerance to various mycotoxins, including DON, without exhibiting significant *in vivo* bioaccumulation [22,23,24]. This characteristic may be attributed to their unique gut microenvironment and highly diverse microbial communities [25,26,27]. Previous studies have further indicated that host enzymes, gut-derived microbial strains, and substrate-associated microorganisms within the BSFL system may all participate in mycotoxin bioconversion or mitigation processes. For instance, the BSFL S9 enzyme fraction can convert a portion of AFB1 into aflatoxicol and aflatoxin P1, the gut-derived strain *Stenotrophomonas acidaminiphila* can enhance AFB1 degradation, and both BSFL and substrate-associated microorganisms have been confirmed to be involved in AFB1 bioconversion [28,29,30]. However, current research on treating DON-contaminated feed with BSFL remains largely focused on larval tolerance and residue distribution. The relationships among larval growth and bioconversion performance, DON distribution between larvae and frass, and gut microbial responses under DON exposure remain insufficiently understood.

Building upon this rationale, the present study used BSFL as an experimental model to evaluate the effects of DON-contaminated feed on larval growth performance, bioconversion efficiency, and DON residue distribution. In addition, 16S rRNA high-throughput sequencing was used to characterize the diversity, taxonomic composition, and differential taxa of the larval gut microbiota under DON exposure. This study aimed to assess the feasibility of using BSFL to process DON-contaminated feed and to provide a theoretical basis for the bioconversion of DON-contaminated feed or cereal by-products, as well as for the future screening of potential functional microbial taxa.

## 2. Materials and Methods

### 2.1. Sample Preparation and Materials

The DON standard used for chromatographic analysis and feed preparation was purchased from Sigma-Aldrich (St. Louis, MO, USA). The basal compound feed and BSF eggs used in this study were obtained from the experimental base of Henan Agricultural University (Zhengzhou, China). The BSF eggs were incubated in an artificial climate chamber at 30 °C and 65% relative humidity, using wheat bran adjusted to approximately 65% moisture as the hatching substrate. After hatching, the neonate larvae were transferred to standard wheat bran and reared for 4 days before being used in the formal experiment. 

### 2.2. Experimental Design and Rearing Management

The experiment was conducted using a completely randomized design with two treatment groups and six replicates per group. Each replicate contained 150 healthy and uniformly sized BSFL. The control group (CON) was provided with 100 g of basal compound feed, whereas the DON-treated group (DON) received 100 g of basal feed supplemented with DON to achieve a final concentration of 1 mg/kg. The substrates in both groups were adjusted with sterile water and thoroughly homogenized. The larvae were then introduced into plastic rearing boxes (8.5 cm × 14 cm) covered with breathable mesh. All boxes were maintained in an environmental chamber for 10 days at 30 °C and 65% relative humidity. During the experiment, larval growth and health status were monitored daily, and sterile water was added as needed according to substrate moisture conditions to maintain appropriate humidity.

### 2.3. Sample Collection and Measurement of Biological Parameters

Before larvae were introduced, initial substrate samples were collected from each group and stored at −20 °C for the determination of DON concentrations. At the end of the rearing period, larvae were thoroughly separated from the frass by sieving. For each replicate, 20 larvae were randomly selected for body length measurement. Another 50 larvae were randomly sampled, rinsed with distilled water, blotted dry with absorbent paper, and weighed to calculate the average individual body weight. After these measurements, the larvae were returned to their respective containers. The total number of surviving larvae in each replicate was recorded, and all frass was collected to determine its fresh weight. For gut microbiota analysis, 50 larvae were randomly selected from each replicate, rinsed with tap water, surface-sterilized by immersion in 75% ethanol for 1 min, and then rinsed twice with sterile water. Gut tissues were dissected under sterile conditions and immediately frozen at −80 °C. Finally, representative subsamples of the initial substrate, harvested larvae, and final frass were collected to determine their moisture content. Moisture content was measured using the direct drying method, in which samples were dried at 105 °C to a constant weight. The dry weights of the substrate, larvae, and frass were then calculated and used for the evaluation of bioconversion-related indicators.

To evaluate the growth performance and bioconversion efficiency of BSFL, four core indicators were calculated at the end of the experimental period: survival rate, substrate reduction rate (SR), waste reduction index (WRI) and efficiency of conversion of digested feed (ECD). The corresponding equations were adapted from Parra-Pacheco et al. [31] and Addeo et al. [32]:

(1) $$\mathrm{Survival}\;\mathrm{Rate}\;(\%)\;=\;\frac{N_{\mathrm{final}}}{N_{\mathrm{initial}}}\;\text{×}\;100$$

(2) $$\mathrm{SR}\;(\%)=\mathrm{SD}W_{\mathrm{initial}}\;-\mathrm{FD}W_{\mathrm{final}}\mathrm{SD}W_{\mathrm{initial}}\;\text{×}\;100$$

(3) $$\mathrm{WRI}\;(\%)=\frac{\mathrm{SD}W_{\mathrm{initial}}-\mathrm{FD}W_{\mathrm{final}}}{\mathrm{SD}W_{\mathrm{initial}}\;\text{×}\;t}\;\text{×}\;100$$

(4) $$\mathrm{ECD}\;(\%)=\frac{\mathrm{LD}W_{\mathrm{final}}-\mathrm{LD}W_{\mathrm{initial}}}{\mathrm{SD}W_{\mathrm{initial}}-\mathrm{FD}W_{\mathrm{final}}}\;\text{×}\;100$$

where N, SDW, FDW, and LDW denote larval number, substrate dry weight, frass dry weight, and larval dry weight, respectively; t represents the larval rearing time (days); the subscripts initial and final indicate values measured before and after larval rearing, respectively.

### 2.4. DON Residue Determination by HPLC

DON content was determined using immunoaffinity column cleanup followed by high-performance liquid chromatography with UV detection (HPLC-UV), based on the method of Iqbal et al. [33] with modifications. Briefly, samples were ground and passed through a 60-mesh sieve. A 5.0 g aliquot was accurately weighed and extracted with 25 mL of 80% methanol-water solution (*v*/*v*) by shaking at 30 °C and 220 rpm for 1 h, followed by ultrasonication for 20 min. The extract was centrifuged at 7000 rpm for 20 min, and the supernatant was filtered through a 0.22 μm membrane filter. Then, 2 mL of filtrate was mixed with 12 mL of phosphate-buffered saline (PBS) and passed through a DON immunoaffinity column at a flow rate of 1–2 drops/s. The column was rinsed with 10 mL of PBS and sequentially eluted with methanol and ultrapure water. The eluates were combined and evaporated to dryness under a nitrogen stream at 50 °C. The residue was reconstituted in 1.8 mL of 10 mmol/L ammonium acetate solution and 0.2 mL of methanol, and then filtered before HPLC analysis. HPLC analysis was performed using a Diamonsil C18 reversed-phase column (4.6 mm × 150 mm, 5 μm) at 35 °C, with detection at 218 nm. The mobile phase consisted of acetonitrile and water (10:90, *v*/*v*) at a flow rate of 1.0 mL/min, and the injection volume was 20 μL.

### 2.5. 16S rRNA Gene Sequencing and Analysis

Larval gut samples were collected in sterile centrifuge tubes and microbial genomic DNA was extracted using the E.Z.N.A.^®^ Soil DNA Kit (Omega Bio-tek, Norcross, GA, USA) according to the manufacturer’s instructions. The V3–V4 hypervariable region of the bacterial 16S rRNA gene was amplified using the universal primers 338F (5′-ACTCCTACGGGAGGCAGCAG-3′) and 806R (5′-GGACTACHVGGGTWTCTAAT-3′). PCR amplification was performed under the following conditions: initial denaturation at 95 °C for 3 min; 27 cycles of denaturation at 95 °C for 30 s, annealing at 55 °C for 30 s, and extension at 72 °C for 30 s; and a final extension at 72 °C for 10 min. PCR products were purified using an AxyPrep DNA Gel Extraction Kit (Axygen Biosciences, Union City, CA, USA) and quantified using a Quantus™ Fluorometer (Promega, Madison, WI, USA). The purified amplicons were subjected to paired-end sequencing on an Illumina MiSeq PE300 platform (Illumina, San Diego, CA, USA). After quality control, raw sequencing reads were processed using UPARSE software (version 7.1). Chimeric sequences were removed, and high-quality sequences were clustered into operational taxonomic units (OTUs) at 97% sequence similarity. Taxonomic classification of representative OTU sequences was performed using the RDP Classifier (version 2.2) against the SILVA v138 database with a 70% confidence threshold [34,35]. Based on the OTU annotation results, alpha diversity indices, Venn diagrams, principal coordinate analysis (PCoA), and microbial community composition at the phylum and genus levels were analyzed using the Majorbio Cloud Platform.

### 2.6. Statistical Analysis

All data were initially compiled using Microsoft Excel. Statistical analyses of growth performance and bioconversion parameters were performed using IBM SPSS Statistics software (version 26.0). Differences between the CON and DON groups were analyzed using independent-samples *t*-tests. All results are expressed as mean ± standard deviation (SD), and differences were considered statistically significant at *p* < 0.05. Graphs were generated using GraphPad Prism software (version 10.1.2) and R software (version 10.0).

For gut microbiota analysis, alpha diversity indices, including Chao1, ACE, Shannon, and Simpson, were compared between the CON and DON groups using the Wilcoxon rank-sum test. Beta diversity was evaluated using principal coordinate analysis (PCoA) based on Bray–Curtis distances, and group differences were assessed using Analysis of Similarities (ANOSIM). Differences in the relative abundance of bacterial taxa at the genus levels were analyzed using a two-sided Wilcoxon rank-sum test, and *p* values were adjusted using the Benjamini–Hochberg false discovery rate (BH-FDR) method. Taxa with FDR-adjusted *p* values < 0.05 were considered statistically significant.

## 3. Results

### 3.1. Growth Performance of BSFL Under DON Exposure

**Figure 1:**
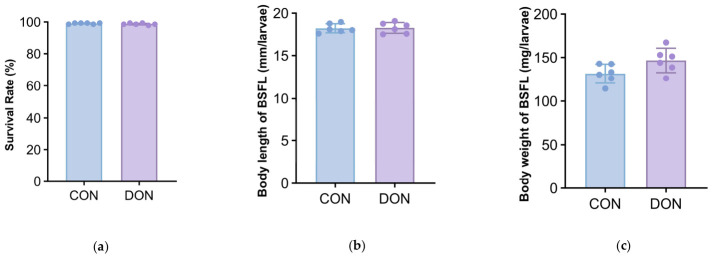
(**a**) Survival rate. (**b**) Body length of BSFL. (**c**) Body weight of BSFL. Data are presented as mean ± SD, with each dot representing one biological replicate (*n* = 6). Differences between the CON and DON groups were analyzed using an independent-samples *t*-test. No significant differences were observed between the two groups for all parameters (*p* > 0.05).

As shown in Figure 1, DON exposure did not significantly affect the growth performance of BSFL. The survival rates in both the CON and DON groups were close to 100%, with no significant difference between the two groups (*p* > 0.05) (Figure 1a). Similarly, larval body length was not significantly altered by DON exposure (Figure 1b). Although larval body weight showed a slight increasing trend in the DON group compared with the CON group, this difference was not statistically significant (*p* > 0.05) (Figure 1c).

### 3.2. Substrate Utilization and Bioconversion Performance Under DON Exposure

**Figure 2:**
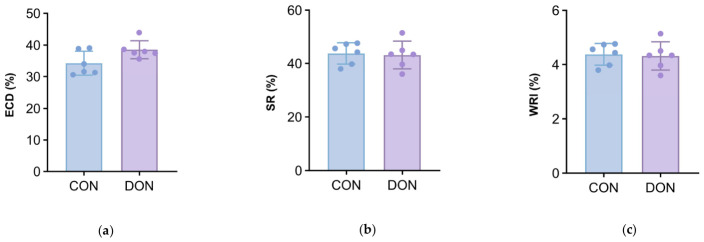
Bioconversion performance of BSFL under DON exposure. (**a**) Efficiency of conversion of digested feed. (**b**) Substrate reduction rate. (**c**) Waste reduction index. Data are presented as mean ± SD, with each dot representing one biological replicate (*n* = 6). Differences between the CON and DON groups were analyzed using an independent-samples *t*-test. No significant differences were observed between the two groups for all parameters (*p* > 0.05).

As shown in Figure 2, DON exposure did not significantly affect the bioconversion performance of BSFL. The ECD showed a slight increasing trend in the DON group compared with the CON group, but the difference was not statistically significant (*p* > 0.05) (Figure 2a). Similarly, no significant differences were observed in SR or WRI between the two groups (*p* > 0.05) (Figure 2b,c).

### 3.3. DON Residues in Substrate, Larvae, and Frass After BSFL Treatment

DON residues in the initial substrate, harvested larvae, and final frass were determined to evaluate the distribution of detectable parent DON after BSFL treatment. As shown in Table 1, DON was below the limit of detection (LOD) in all substrate, larval, and frass samples from the CON group, indicating that no detectable DON contamination occurred in the control treatment. In the DON group, the DON concentration in the initial substrate was 961.39 ± 19.22 μg/kg. After 10 days of BSFL treatment, DON was below the LOD in harvested larval samples, suggesting that detectable parent DON did not accumulate in the larval body under the present experimental conditions. Meanwhile, DON concentration in the frass was 181.76 ± 3.00 μg/kg, which was approximately 81.10% lower than that in the initial substrate, indicating an apparent decrease in detectable parent DON during BSFL treatment.

### 3.4. Gut Microbial Responses of BSFL to DON Exposure

#### 3.4.1. Sequencing Depth and OTU Distribution of the BSFL Gut Microbiota Under DON Exposure

**Figure 3:**
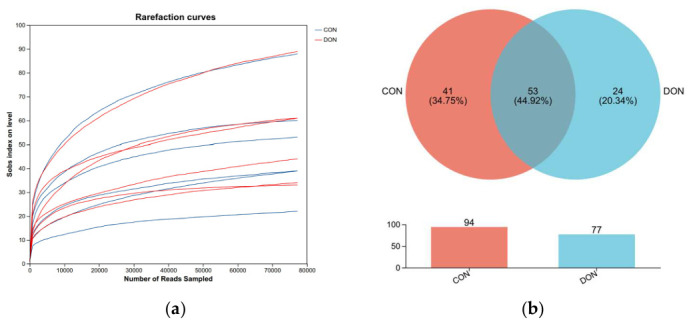
Sequencing depth and OTU distribution of the BSFL gut microbiota under DON exposure. (**a**) Rarefaction curves of gut microbial communities. (**b**) Venn diagram showing shared and unique OTUs between the CON and DON groups.

As shown in Figure 3a, the rarefaction curves of all samples gradually reached a plateau as the number of sequencing reads increased, indicating that the sequencing depth was sufficient to capture the gut microbial communities of BSFL and was adequate for subsequent analyses. The Venn diagram showed that 53 operational taxonomic units (OTUs) were shared between the CON and DON groups, accounting for 44.92% of the total OTUs. In addition, 41 OTUs (34.75%) and 24 OTUs (20.34%) were unique to the CON and DON groups, respectively (Figure 3b). The number of unique OTUs was lower in the DON group than in the CON group.

#### 3.4.2. Effects of DON Exposure on Gut Microbial Alpha Diversity of BSFL

**Figure 4:**
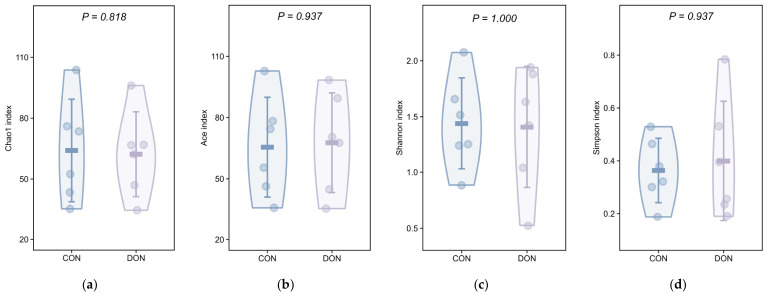
Effects of DON exposure on the gut microbial alpha diversity of BSFL. (**a**) Chao1 index. (**b**) ACE index. (**c**) Shannon index. (**d**) Simpson index. Each point represents one biological replicate. Differences between the CON and DON groups were analyzed using the Wilcoxon rank-sum test, and the corresponding *p* values are shown in each panel. No significant differences were observed between the two groups for all alpha diversity indices (*p* > 0.05).

As shown in Figure 4, DON exposure did not significantly affect the gut microbial alpha diversity of BSFL. Compared with the CON group, the DON group showed no significant differences in the Chao1 or ACE indices, indicating that microbial community richness was not markedly altered. Similarly, no significant differences were observed in the Shannon or Simpson indices, suggesting that microbial community diversity and evenness remained relatively stable under DON exposure. The Wilcoxon rank-sum test confirmed that all alpha diversity indices were not significantly different between the CON and DON groups (*p* > 0.05).

#### 3.4.3. Effects of DON Exposure on Gut Microbial Beta Diversity of BSFL

**Figure 5:**
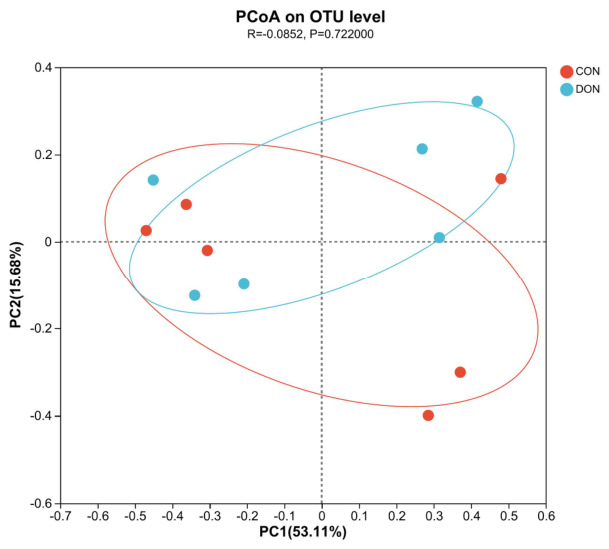
Principal coordinate analysis (PCoA) of the gut microbiota of BSFL under DON exposure. The PCoA plot was generated based on Bray-Curtis distances at the OTU level, with each point representing one biological replicate. PC1 and PC2 explained 53.11% and 15.68% of the total community variation, respectively. Group differences in overall microbial community structure between the CON and DON groups were evaluated using Analysis of Similarities (ANOSIM). No significant difference was observed between the two groups (*R =* −0.0852, *p* = 0.722).

To further evaluate differences in the overall gut microbial community structure, beta diversity was analyzed using principal coordinate analysis (PCoA) based on Bray-Curtis distances at the OTU level (Figure 5). In the PCoA plot, the CON and DON groups showed visible overlap, indicating that the overall gut microbial communities were not clearly separated between treatments. The first two principal coordinates, PC1 and PC2, explained 53.11% and 15.68% of the total community variation, respectively. Further statistical evaluation using Analysis of Similarities (ANOSIM) confirmed that DON exposure did not significantly alter the overall gut microbial community structure of BSFL (*R* = −0.0852, *p* = 0.722). These results suggest that DON exposure at 1 mg/kg did not markedly disrupt the beta diversity of the BSFL gut microbiota under the present experimental conditions.

#### 3.4.4. Effects of DON Exposure on Gut Microbial Community Composition of BSFL

**Figure 6:**
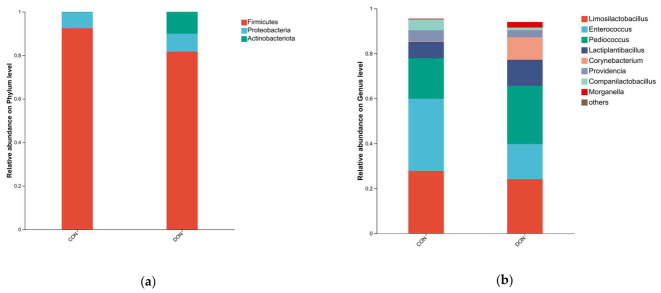
Gut microbial community composition of BSFL under DON exposure. (**a**) Relative abundance of gut microbiota at the phylum level. (**b**) Relative abundance of gut microbiota at the genus level.

As shown in Figure 6, *Firmicutes* was the dominant phylum in both the CON and DON groups. At the genus level, *Limosilactobacillus*, *Enterococcus*, and *Pediococcus* were the predominant taxa in the CON group. In the DON group, numerical changes in the relative abundance of several genera were observed, including decreases in Enterococcus and increases in *Pediococcus*, *Lactiplantibacillus*, and *Corynebacterium*. However, differential abundance analysis using a Wilcoxon rank-sum test with Benjamini–Hochberg FDR correction showed that none of the genera exhibited statistically significant differences between the CON and DON groups (FDR-adjusted *p* > 0.05; Supplementary Table S1).

## 4. Discussion

### 4.1. Growth Tolerance of BSFL Under DON Exposure

The growth performance results showed that DON exposure did not significantly affect the survival rate, body length, or body weight of BSFL, indicating that the larvae were able to maintain normal growth in DON-contaminated substrates under the experimental conditions. Although DON can induce adverse toxicological responses in conventional livestock and poultry, including reduced feed intake, intestinal injury, immune dysfunction, and oxidative stress [1,36,37], BSFL, as saprophagous insects, have a strong capacity to adapt to complex organic substrates. This finding is consistent with previous studies reporting that BSFL can tolerate various mycotoxins, including DON and AFB1, with limited evidence of *in vivo* bioaccumulation [22,23,24,38]. Notably, larval body weight in the DON group showed a slight increasing trend compared with the CON group, but the difference was not statistically significant (*p* > 0.05). Therefore, this trend should not be interpreted as a direct growth-promoting effect of DON. Rather, it suggests that DON exposure under the present experimental conditions did not noticeably inhibit individual larval growth. Similarly, Xiao et al. [38] reported that BSFL could maintain relatively normal biomass accumulation under higher DON exposure, whereas significant reductions in crude protein and lipid yields occurred only at higher exposure levels. Overall, these results further support the tolerance of BSFL to DON-contaminated substrates, although the underlying adaptive responses require further investigation in combination with gut microbiota analysis.

### 4.2. Effects of DON Exposure on the Bioconversion Performance of BSFL

From the perspective of bioconversion performance, DON exposure did not impair the overall substrate utilization capacity of BSFL under the experimental conditions. In the present study, no significant differences were observed in ECD, SR, or WRI between the CON and DON groups. These results suggest that DON exposure did not reduce larval substrate conversion or waste reduction capacity. BSFL are known for their strong adaptability to complex organic substrates and their capacity to convert low-value organic residues into insect biomass rich in proteins and lipids [39,40,41]. Combined with the growth performance results, the absence of significant changes in larval survival, body length, and body weight further indicates that DON-contaminated feed did not cause obvious detrimental effects on larval growth or overall bioconversion performance. However, the safe utilization of BSFL-derived products depends not only on larval growth and conversion efficiency but also on DON residue distribution and potential bioaccumulation risks in larvae and frass. Therefore, DON residue analysis is essential for a more comprehensive evaluation of the safety of this BSFL-based bioconversion system.

### 4.3. DON Residues During BSFL Bioconversion

Residue analysis showed that DON was below the limit of detection (LOD) in larval samples after BSFL treatment, while its level in the frass was approximately 81.10% lower than that in the initial substrate. This result indicates that DON did not detectably accumulate in BSFL larvae and that BSFL treatment markedly reduced the residual DON level associated with the final frass fraction.

Regarding residue distribution, the absence of detectable DON accumulation in larvae is consistent with previous studies on BSFL reared on mycotoxin-contaminated substrates. Previous studies have shown that DON generally does not persistently accumulate in BSFL after treatment of DON-contaminated substrates, but is more likely to remain in the residual substrate or frass fraction. Camenzuli et al. [22] reported that only 39–55% of DON could be recovered during BSFL treatment, suggesting that part of the DON may not remain in larvae or residues as the parent compound. Similarly, Gulsunoglu et al. [23] observed that DON levels in larvae reared on Fusarium-contaminated wheat became negligible at later stages, whereas DON concentrations in the residual feed did not consistently decrease. This indicates that the final distribution of DON may be influenced by substrate composition, contamination source, and larval feeding activity. More recently, Ivanova et al. [42] reported that, under higher DON exposure, DON was mainly retained in the frass or residual substrate at the end of the experiment, with only low levels detected in larvae.

Together, these findings indicate that DON fate during BSFL treatment may involve multiple processes, including feeding-related dilution, retention in residual substrate, excretory transfer, and potential microbial transformation, rather than simple larval bioaccumulation. In the present study, the reduced DON level in frass and the absence of detectable DON in larvae support the potential of BSFL for processing DON-contaminated substrates with limited risk of larval toxin accumulation. These results provide a basis for further exploring bioconversion using BSFL as a sustainable strategy for managing mycotoxin-contaminated feed resources.

### 4.4. Gut Microbial Diversity and Community Stability Under DON Exposure

DON exposure did not significantly affect the overall diversity and community structure of the BSFL gut microbiota, as indicated by the alpha and beta diversity analyses. This suggests that the gut microbial community maintained a relatively stable ecological structure under the present experimental conditions.

Previous studies have reported that the gut microbiota of BSFL has strong substrate responsiveness and functional plasticity, allowing it to participate in organic matter degradation, nutrient conversion, and host homeostasis maintenance in complex waste or contaminated substrate environments [43,44,45,46]. Therefore, the pattern observed in the present study, namely stable alpha diversity together with minor compositional variability, may reflect a stable gut microbial community accompanied by subtle taxonomic changes under DON exposure.

When considered together with the high larval survival rate, stable growth performance, and DON levels below the LOD in larval samples, this gut microbial modulation may help explain how BSFL maintained growth and substrate utilization in DON-contaminated substrates. These results suggest that the BSFL gut microbiota remained relatively stable while undergoing overall community stability, which may contribute to host adaptation and tolerance under DON exposure.

### 4.5. Compositional Trends in Gut Microbiota Under DON Exposure and Their Potential Functional Significance

Although slight shifts in the relative abundance of specific bacterial taxa were observed, differential abundance analysis indicated that these changes were not statistically significant after Benjamini–Hochberg FDR correction. This suggests that DON exposure did not induce a robust restructuring of the gut microbial community, but rather was associated with minor compositional variability within a generally stable microbial ecosystem. The lack of significant differences at the genus level is consistent with both the alpha and beta diversity results, indicating that the overall gut microbial community structure of BSFL remained stable under the present experimental conditions.

At the taxonomic composition level, Firmicutes remained the predominant phylum in both the CON and DON groups, indicating that it may represent a core component of the BSFL gut microbiota under the present experimental conditions. This finding is consistent with previous reports showing that *Firmicutes* is commonly dominant in the BSFL gut [47,48]. Members of Firmicutes, including Enterococcus, Bacillus, Lactobacillus, *Pediococcus*, and *Lactiplantibacillus*, are often associated with carbohydrate fermentation, organic acid production, and nutrient conversion, which may be related to substrate digestion and gut environmental stability in BSFL.

In the present study, several lactic acid bacteria-related genera showed numerical changes in relative abundance. Enterococcus showed a lower relative abundance in the DON group, whereas *Pediococcus* and *Lactiplantibacillus* showed higher relative abundance. These observations indicate subtle taxonomic variability under DON exposure, but they should not be interpreted as statistically significant shifts because no genus remained significant after FDR correction. Some lactic acid bacteria have been reported to possess mycotoxin-binding or detoxification potential. For example, Yao et al. [49] reported that Lactobacillus rhamnosus MY-1 removed DON through both biosorption and biodegradation, with a removal rate of 93.34% after 48 h.

Previous studies have shown that DON can be transformed into less toxic metabolites, such as DOM-1 and 3-epi-DON, through microbe-mediated pathways, including de-epoxidation and epimerization [1,17,50]. In this context, the compositional trends observed in the BSFL gut microbiota may reflect microbial adaptation associated with substrate utilization, gut environmental stability, and host–microbe interactions under DON exposure. Together with the stable alpha and beta diversity results, these findings suggest that the BSFL gut microbiota maintained community-level stability while exhibiting subtle taxonomic variability, which may support larval adaptation and stable bioconversion performance during exposure to DON-contaminated substrate.

## 5. Conclusions

This study demonstrated that BSFL were able to maintain normal growth and selected bioconversion indicators when reared on DON-contaminated feed. DON exposure had no significant effect on larval survival, body length, body weight, efficiency of conversion of digested feed, substrate reduction rate, or waste reduction index. Residue analysis showed that DON was below the limit of detection in larval samples after BSFL treatment, while the DON level in frass was approximately 81.10% lower than that in the initial substrate. This result indicates that BSFL treatment contributed to a marked reduction in DON residues during bioconversion, without detectable DON bioaccumulation in larvae. Meanwhile, gut microbiota analysis showed that DON exposure did not significantly affect alpha or beta diversity, and no genus-level taxa differed significantly after Benjamini–Hochberg FDR correction. Together with the stable growth performance, unchanged bioconversion indicators, absence of detectable DON accumulation in larvae, and reduced DON residues in frass, these findings support the potential of BSFL for processing DON-contaminated feed while maintaining gut microbial stability.

## Figures and Tables

**Table 1:** DON residues in the initial substrate, harvested larvae, and final frass after BSFL treatment.

Sample	Substrate	Larvae	Frass
CON	<LOD	<LOD	<LOD
DON	961.39 ± 19.22	<LOD	181.76 ± 3.00

Note: Values are expressed as mean ± SD (*n* = 6). CON, control group; DON, DON-treated group; LOD, limit of detection. The LOD for DON was 100 μg/kg. Samples below the LOD are presented as <LOD.

## Data Availability

The original contributions presented in this study are included in the article and Supplementary Material. Further inquiries can be directed to the corresponding authors.

## Supplementary Materials

The following supporting information can be downloaded at: https://www.mdpi.com/article/10.3390/microorganisms14071452/s1, Table S1: Differential abundance analysis of gut microbiota at the genus level between the CON and DON groups.
